# ROS and Ions in Cell Signaling during Sexual Plant Reproduction

**DOI:** 10.3390/ijms21249476

**Published:** 2020-12-13

**Authors:** Maria Breygina, Ekaterina Klimenko

**Affiliations:** Department of Plant Physiology, Biological Faculty, Lomonosov Moscow State University, 119991 Moscow, Russia; kleo80@yandex.ru

**Keywords:** pollen germination, pollen tube growth, ROS, ions, plant reproduction

## Abstract

Pollen grain is a unique haploid organism characterized by two key physiological processes: activation of metabolism upon exiting dormancy and polar tube growth. In gymnosperms and flowering plants, these processes occur in different time frames and exhibit important features; identification of similarities and differences is still in the active phase. In angiosperms, the growth of male gametophyte is directed and controlled by its microenvironment, while in gymnosperms it is relatively autonomous. Recent reviews have detailed aspects of interaction between angiosperm female tissues and pollen such as interactions between peptides and their receptors; however, accumulated evidence suggests low-molecular communication, in particular, through ion exchange and ROS production, equally important for polar growth as well as for pollen germination. Recently, it became clear that ROS and ionic currents form a single regulatory module, since ROS production and the activity of ion transport systems are closely interrelated and form a feedback loop.

## 1. Introduction

Reactive oxygen species (ROS) in plant tissues are a universal regulatory element associated with various signaling systems, such as phospholipids, calcium, and ROP (Rho of plants) GTPases. The participation of ROS in intercellular cross-talk and morphogenesis at the cellular level, for example, during stomatal movements, zygote polarization, and root hair growth, has been convincingly shown [1,2,3,4].

Sexual reproduction in plants has been extensively studied since this area has both fundamental and practical significance. Most of the data on male gametophyte germination was obtained in vitro, since pollen cultivation is an accessible and convenient technique that allows one to simplify the experimental system. However, recently, many studies have focused on the interaction (1) between gametophytes and (2) of male gametophyte with female tissues of sporophyte [5,6,7]. In this case, regulatory factors found in vitro are tested for in vivo efficacy, mainly using genetic approach and improved fluorescence techniques. A lot of attention is paid to the interactions of peptides with their receptors which provide the control of pollen tube growth by female tissues [8]. However, the accumulated evidence suggests also low-molecular communication between sporophyte tissues and pollen, in particular, through ion exchange and production of ROS [6,7]. The ability of pollen grains to respond to changes in ionic environment was discovered around 45 years ago [9], and recently, it became clear that ROS and ionic currents form a single regulatory system, since ROS production and the activity of ion transporters are tightly interrelated [10,11]. 

## 2. Pollen Germination

### 2.1. ROS Production as an Early Event during Germination 

When a dry, dormant pollen grain lands on a stigma or on the scales of a female cone, it rehydrates and gradually switches to active metabolism [12]. One of the first physiological changes, apparently, is ROS production, since NBT staining reveals ROS in the aperture area already after 5 minutes of in vitro pollen incubation in kiwi [13]; staining of non-germinated tobacco pollen grains with ROS-sensitive dyes displays their apoplastic and mitochondrial localization [14]. ROS production has also been described in non-germinated pollen grains of Arizona cypress [15]. Mitochondrial ROS production must be tightly controlled, as its excess, for example, in defective pollen leads to cell death and, as a consequence, to cytoplasmic male sterility (CMS) [16]. The involvement of ROS in pollen abortion has been reported for many cytoplasm male sterile crop varieties, for example, cotton [17], pepper [18], and rice [19]. In these cases, excessive ROS production was associated with reduced abundance of superoxide dismutase (SOD), ascorbate peroxidase (APX), catalase [19], and peroxisomal-like protein [17] in mitochondria.

Besides their generation in mitochondria, ROS in pollen are produced on the plasma membrane of vegetative cell and can be accumulated in the apoplast. ROS release from pollen grains in vitro was recorded after 20 minutes in tobacco and kiwi [13,14] and after 10 min in spruce [20]. In these cases, ROS production was associated with NADPH oxidase, since its inhibitor DPI (diphenyl iodonium) suppressed ROS accumulation in the germination medium [14,20] and ROS synthesis in pollen grains [13,15]. In *Arabidopsis* pollen, two isoforms of plasma membrane NADPH oxidase are involved in ROS synthesis: RbohH and RbohJ (respiratory burst oxidase protein homolog) [21]. Cytochemical analysis of pollen grains germinating on stigma showed that double mutants *rbohH,J* lack H_2_O_2_ accumulation in the apoplast, which is typical for wild-type pollen [22]. SOD and MnTMPP (ROS quencher that mimics the activity of SOD and catalase) severely reduce pollen germination efficiency in blue spruce [20]. A negative effect has been also reported for cypress [15]. In angiosperms, the situation is, apparently, more complicated than in conifers since the balance of ROS production/elimination largely depends on pistil tissues. Thus, in tobacco, low concentrations of ascorbic acid and DPI reduce ROS content in pollen, but stimulated germination as well as MnTMPP [23]. This data indicates that the level of ROS produced by tobacco pollen is excessive relative to optimal. However, no such effect was observed in kiwi; all investigated concentrations of antioxidants blocked pollen germination [13]. Taken together, the facts described indicate the importance of endogenous ROS and, in particular, NADPH oxidase-derived ROS during initial germination stages in both angiosperms and conifers.

During in vivo germination in *Arabidopsis*, ROS synthesis is very important already at the stage of pollen grain hydration [24]. Snf1(sucrose nonfermenting 1)-related kinase1 protein kinase complex belongs to a family of highly conserved serine/threonine kinases and is involved in pollen, embryo, seedling and organ development regulation as well as in sugar, stress and hormonal signaling. It turned out that the mutant lacking subunit KINβγ of SnRK1 protein complex simultaneously exhibit a reduced level of endogenous ROS and hydration disorders, which did not appear in vitro (when water was in excess), as well as when water was added on the stigma. The same disturbances were observed upon overexpression of catalase, which drastically reduced the level of endogenous H_2_O_2_ in pollen [24]. Thus, a relationship was revealed between ROS production at the early germination stage and water flow into dry pollen grains.

### 2.2. Changes of Ionic Status during Early Germination Stages 

In parallel with ROS production, the exact time of which has not been established, since it may differ for different species, active protein synthesis begins, and pollen starts the preparation for polar growth, which includes significant changes in ionic homeostasis. One of the earliest events is the release of anions from pollen grains [25]; in tobacco it begins within 2 minutes of incubation. Other early changes include cytoplasmic pH shift towards alkaline values and hyperpolarization of the plasmalemma [26,27], in tobacco they occur simultaneously. Experiments with proton pump inhibitor and activator demonstrate significance of this enzyme in triggering the early germination stages, apparently due to the cytoplasm alkalization [27,28,29,30]. The H^+^-ATPase can also take part in plasma membrane hyperpolarization, together with anion channels: suppression of their activity blocks membrane potential (MP) shift in tobacco pollen [25].

When metabolism activation is completed, pollen grain undergoes polarization and segregation of the germination pole (cytoplasmic zone where a pollen tube will form). This domain differs from other zones in the arrangement of organelles: clusters of vesicles and mitochondria, which will provide the delivery of membrane material and energy for pollen tube growth, are accumulated at the germination pole [31]. However, some time before the polar growth is launched and cytoplasm protrusion becomes noticeable, physiological segregation of the pole occurs, for which ionic currents are essential. In early studies, electric currents crossing lily pollen grain were recorded, which, apparently, set the polarity for future germination [9,32]. On *Arabidopsis* pollen, it was shown that 9-15 minutes after in vivo pollination, a local increase in the intracellular calcium concentration begins in the germination pole [33]; it apparently causes NADPH oxidase activation and subsequent local ROS synthesis [21].

### 2.3. ROS Production on Stigma 

ROS synthesis and ion currents at the early germination stage are interrelated and can stimulate each other forming a regulatory feedback loop; however, this system may involve not only endogenous ROS (synthesized by pollen) but also exogenous ROS coming from the sporophyte tissues. A current hypothesis states that ROS synthesis should be considered, inter alia, as a way of activating and/or supporting pollen germination by female tissues.

ROS are synthesized on stigmas of various (if not all) flowering plants; in total, more than 20 species from different families have been studied to date, and this property is common for all studied species [5,34,35,36]. According to inhibitory experiments, the main ROS on stigma is hydrogen peroxide [35,36]. The presence of various ROS-regulating enzymes, in particular, peroxidases [37,38], on stigma and in stigma exudate has been widely accepted. On the other hand, in vitro pollen grain diffusates caused the inhibition of peroxidase activity [39]. Thus, the final balance between ROS production and elimination during in vivo germination in flowering plants is the result of a complex interaction between sporophyte and male gametophyte, which includes both low molecular weight components and antioxidant enzymes. 

### 2.4. Perception of Exogenous ROS Signal by Pollen

Moderate H_2_O_2_ concentrations activate pollen germination in tobacco, while high concentrations inhibit [23]; for kiwi, high peroxide concentrations also have an inhibitory effect [13]. In spruce, the latter effect did not appear - the presence of ROS in a wide concentration range (0.1–2 mM) does not reduce the germination efficiency [20]; So far, it can be assumed that during pollen germination in gymnosperms, female tissues (cones) do not produce noticeable amounts of superoxide radical or peroxide, and only endogenous ROS are used to activate ion currents and other physiological effects in pollen. To confirm or disprove this hypothesis, one needs to find out if there are ROS in female cones before and during pollination.

One of the functions performed by ROS in plant cell is the control of cell wall cytomechanics. Moreover, while some ROS loosen the extracellular matrix, others, on the contrary, promote cross-linking of polymers [40]. Wall loosening can occur by non-protein-mediated scission of polysaccharides through •OH attack [41], while H_2_O_2_ can strengthen polymers via peroxidase-mediated cross-linking of hydroxycinnamates [42]. As shown in tobacco, this function is critical for pollen grains: a shift in ROS balance leads to impaired pollen germination. Hence, pollen grains treated with an excess of •OH were unstable to hypotonic stress and burst, and those treated with high concentrations of H_2_O_2_ became too hard and could not launch polar growth although remained viable [23]. For gymnosperms, ROS-mediated regulation of cell wall stiffness determines not only the germination efficiency, but also the pattern of pollen tubes appearance: in many species of the Pinaceae family, two tubes can appear from one pollen grain [43,44], which, as it turned out, depends not on the availability of nutrients, but mainly on the properties of the pollen wall regulated by ROS [43].

However, ROS functions are not limited to the cell wall. Exogenous ROS, affecting pollen in flowering plants, can specifically activate ion channels: in protoplasts from lily pollen grains, calcium and potassium currents are stimulated by H_2_O_2_ (100 μM) [45]; in pear pollen protoplasts 10 mM H_2_O_2_ activates the calcium current [46]. For tobacco, similar results were obtained in different concentration range: intracellular [Ca^2+^] was assessed by a fluorescent method, and it clearly reacted to peroxide already at 10 μM. The effect was abolished by calcium channel inhibitor nifedipine [47]. Another important effect was the plasma membrane hyperpolarization in H_2_O_2_-treated protoplasts (10 μM) [47].

Thus, for angiosperms (although we can speak with confidence only of a few species), ROS, and in particular H_2_O_2_, are an important product of female sporophyte tissues enhancing germination, causing membrane hyperpolarization, activation of calcium currents, and, possibly, other more delayed effects.

## 3. Pollen Tube Growth

The pollen tube of angiosperms is characterized by an extremely rapid growth, which is supported by physiological and structural zoning of the cytoplasm [48]. It is generally accepted to distinguish apical, subapical, and distal domains in the pollen tube; the distal, in turn, has its own subdivisions, based on the presence of certain organelles in it. The segregation of cytoplasmic zones is maintained due to a set of regulatory mechanisms, including small GTPases and signaling phospholipids, which are highlighted in a recent review [49]; here, we focus on those that have become the subject of the present review.

Endogenous mechanisms maintaining the polar growth are conveniently studied in vitro, but female sporophyte tissues produce a number of their own molecules that can influence tube growth by enhancing, directing, or blocking it. In particular, the direction of tube growth can be affected by [Ca^2+^], NO, and ROS in pistil tissues, and the latter can both support growth and stop it if fertilization is undesirable [7,50,51].

### 3.1. Ionic Homeostasis and ROS Production in Growing Pollen Tube

**Figure 1:**
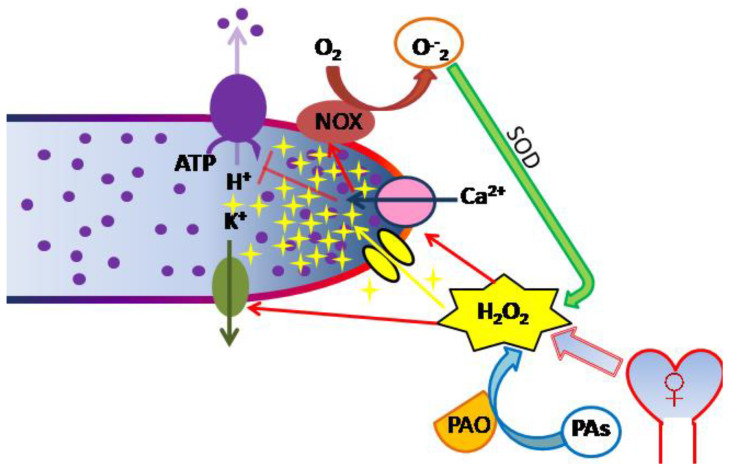
ROS and ion homeostasis in the regulation of pollen tube growth (simplified diagram). Three sources of apoplastic ROS during pollen tube growth are shown: NOX in the plasma membrane produces superoxide radical, SOD dismutates it to hydrogen peroxide; PAO produces H_2_O_2_ during polyamine oxidation; female tissues of the sporophyte produce ROS that control pollen tube growth in vivo. Hydrogen peroxide enters the pollen tube through aquaporins and forms apical ROS accumulation (yellow stars). The main targets for apoplastic ROS are shown: Ca²⁺-permeable ion channels and K^+^-permeable ion channels are activated by H_2_O_2_, lateral membrane potential is affected (apex is red (depolarized), shank is blue (hyperpolarized)). Apical calcium gradient in the cytoplasm is shown in blue color. High [Ca^2+^] reduces the proton pump activity, which forms the alkaline band (protons shown as circles). NOX—NADPH-oxidase, SOD—superoxidedismutase, PAO—polyamineoxidase, Pas—polyamines.

The uneven distribution and activity of ion transporters cells with polar growth results in gradients of ion concentrations and membrane potential (MP) corresponding to different cell domains (Figure 1). Thus, [Ca^2+^] is highest in the apical domain [52]; pH—in subapical; concentration of anions—20 to 50 µm from the tip (*Arabidopsis thaliana*) [53]. MP has the lowest (relatively depolarized) value at the tip; further along the tube length, hyperpolarization is observed [20,54,55]. At the moment, the gradient has already been described in pollen tubes of both flowering plants (tobacco, lily) and conifers (spruce). According to inhibitory analysis, at least H^+^-ATPase and anion channels take part in its maintenance, and for spruce potassium and calcium channels are also involved. Some of these systems are sensitive to endogenous and exogenous ROS, since the gradient changes shape in the presence of both H_2_O_2_, antioxidants, and DPI [20,55]. Interestingly, the sensitivity of pollen tubes to hydrogen peroxide is higher in spruce than that in lily: in spruce, depolarization in subapical region was observed already at 100 μM H_2_O_2_, while in lily the effect was observed at 500 μM, and it was hyperpolarizing. A decrease in the endogenous ROS level in both cases led to hyperpolarization and dissipation of the gradient. Considering these data together with the results obtained on protoplasts from tobacco pollen tubes [47], we can conclude that MP is an indicator with high sensitivity to H_2_O_2_, which means that systems responsible for maintaining the MP gradient can respond to both endogenous ROS, at least partially generated by NADPH oxidase, and ROS produced by female sporophyte tissues.

In growing pollen tubes, pH gradient is maintained: pH at the tip is acidic, in the subapical zone it rises to alkaline values due to H^+^-ATPase activity, and along the tube shank, pH is close to neutral [55,56,57] (Figure 1). pH gradient is affected by significant alterations in ROS production/elimination balance: in MnTMPP-treated in lily pollen tubes pH shifts towards more alkaline values, wherein the difference between apical and subapical zones is leveled [55]; during short-term exposure to 1 mM H_2_O_2_ the alkaline band disappears, the gradient is also leveled; lower concentrations do not affect pH [55]. The authors suggest that the observed effect in H_2_O_2_-treated tubes can be explained by the suppression of H^+^-ATPase activity by high [Ca^2+^], since peroxide stimulates calcium channels [45] (Figure 1). Thus, ROS directly or indirectly regulate the proton pump activity in lily pollen tubes and maintain gradient pH distribution in the cytoplasm.

In all studied pollen tubes, there is a gradient of intracellular calcium concentration: in flowering plants, apical [Ca^2+^] is two orders of magnitude higher than that in the shank [52,58]; gymnosperms have a flatter gradient [59]. ROS production is important for maintaining normal calcium homeostasis: in lily tubes, MnTMPP, even at low concentrations, caused a decrease in calcium concentration and dissipation of [Ca^2+^] gradient [55]. 

Downstream of [Ca^2+^] and pH is the structure and dynamics of actin cytoskeleton controlled by numerous proteins [60]. Among Ca^2+^-dependent actin-binding proteins, for example, profilin, LIM domain-containing proteins, ROP-interactive and CRIB motif-containing protein1 (RIC1), and villins should be mentioned [61]. High [Ca^2+^] in the tube tip supports actin remodeling and ensures its existence in the form of sparse short filaments while low [Ca^2+^] in the shank area correlates with rather thick and stable actin cables; the main mediators in this relationship are proteins from villin/gelsolin/fragmin superfamily [60,61]; subapical actin fringe is tightly associated with the alkaline band, presumably, via actin depolimerizing factor (ADF) and actin-interacting protein 1 (AIP1) [62,63]. Thus, by provoking Ca^2+^ influx in the pollen tube tip and regulating intracellular pH, ROS can thereby affect the actin cytoskeleton.

Since, as has been revealed in protoplasts, calcium transport is closely related to ROS production, one might assume that ROS are also unevenly distributed in the pollen tube. This has been shown by different methods for *Arabidopsis* [64,65], *Pyrus* [66], cypress [15], and two *Picea* species [20,67]. For spruce, it was shown that H_2_O_2_ accumulates in the tube apex, apparently coming from the apoplast (where NADPH oxidase and SOD work) (Figure 1), and in amyloplasts, while most of the O^•^_2_^−^ is produced in mitochondria, and the localization of the two ROS does not coincide [20]. 

ROS are produced in pollen tubes by RbohH and RbohJ. Both proteins located on the pollen tube plasma membrane have EF-hands in their structure and are activated upon binding of calcium ion [21]. In tobacco, transfection with NOX-specific antisense oligodeoxynucleotides (ODNs) resulted in pollen tube growth inhibition, which was rescued by exogenous H_2_O_2_ [68]. In *Arabidopsis*, *rbohH,J* mutants have severe reproductive disorders. The mutant’s pollen has inhibited tube growth and impaired calcium homeostasis [21,69]. Interestingly, mutants for calcium channel genes *cngc7,8* (cyclic nucleotide gated, non-selective, Ca²⁺-permeable ion channels) have phenotypes almost identical to *rbohH,J*, which indicates the feedback regulation of these systems [7]. The features of the *rbohH,J* mutants were revealed in vivo: in wild-type *Arabidopsis* plants, apoplastic ROS production in the area between the pollen tube surface and stigma papilla was detected by histochemistry [22]. In mutants, ROS did not accumulate; growth was impaired. Thus, endogenous ROS produced by NADPH oxidase are essential for pollen tube growth in vitro and in vivo.

However, this enzyme is not the only source of ROS in the male gametophyte: polyamine oxidase (PAO) in pollen can synthesize H_2_O_2_ from polyamines [70] (Figure 1). In *Oryza sativa* seven PAO isoforms have been identified, and one of these, OsPAO7, is specifically expressed in anthers; OsPAO7 produces H_2_O_2_ about 100 times more efficiently than other PAO isoforms [71]. In a recent study the relationship between polyamines and H_2_O_2_ in *Arabidopsis* pollen tubes was reported [65]. Such a relationship had been previously shown for pear: the gradients of total ROS and spermidine in these tubes coincided, and in spermidine-treated tubes (100 µM) cytoplasmic level of these substances increased consistently [66]. It is to be noted that the same exogenously applied polyamines had different effects on the NO/ROS levels pollen grains and tubes. Furthermore, recent studies indicate that PAs regulate pollen germination primarily via regulating the ROS level, while tube elongation primarily influencing the NO level [72].

Polyamines can affect ion homeostasis in plant cells, in many cases, in an indirect manner, with ROS formation as an intermediate stage [70,72,73]. For example, upon treatment with 100 μM spermidine, pear pollen tubes responded with a rapid [Ca^2+^]_cyt_ increase, pH gradient alterations, and switch of growth pattern [74]. The data on polyamine-induced changes in ion transport in root cells is much more plentiful: H^+^-ATPase pumping activity was affected in several species, including both activation (in rice and wheat) and inhibition (in maize) [73]. In some cases, polyamines can cause pollen damage through excessive ROS formation, followed by activation of the antioxidant machinery, degradation of nuclear DNA, and finally, cell death [66].

An ABC transporter carrying polyamines is involved in forming the ROS gradient early during polar growth: short tubes of *atabcg28* (ATP-binding cassette G28) mutant lack the gradient of both total ROS and hydrogen peroxide with maximum at the tip, typical for wild type tubes [65]. It should be noted, however, that total ROS level in this pollen is high, and normal tubes are not formed. 

According to colocalization experiments with both fluorescence microscopy and TEM, mitochondria in the pollen tube are also a source of ROS [20,75,76]; however, the role of mitochondria-derived ROS in the regulation of polar growth is still questionable. To date, their reduced production has been associated with loss of mitochondrial function in self-incompatible pear pollen tubes [76].

### 3.2. ROS are Involved in Signal Perception and Mediate Pollen Tube Rupture

Signals that determine pollen tube growth direction include sporophyte-derived ROS, NO, polyamines, and peptides [6,77,78]; in many of these cases, ROS are also involved in signal perception [50].

In tobacco pollen tubes, polyamines applied at a low concentration (10 μM) affected pollen elongation differentially [72]: putrescine negatively regulated pollen tube elongation; spermidine enhanced it, spermine had no effect on pollen tube growth. This influence of polyamines correlated well with their effect on ROS and/or NO levels in pollen tube tip: high NO and low ROS levels in the tip region of treated pollen tubes promoted while the opposite inhibited growth [72].

Recently, ROS was found to be involved in a signaling cascade triggered in response to a peptide signal (RALF4—RAPID ALKALINIZATION FACTOR 4), which is the pistil-side control of pollen tube growth. Exposure of tubes growing in vitro to this peptide caused a sharp increase in ROS level, growth stimulation and prevented tube rupture [79]. On the contrary, H_2_O_2_ quenchers potassium iodide and sodium pyruvate inhibited pollen tube growth and caused rupture. In plant lines that do not produce LORELEI-LIKE GPI-ANCHORED PROTEINS 2/3, involved in the perception of RALF signal, ROS level was significantly reduced, and growth was impaired. The addition of exogenous H_2_O_2_ partially restored these disturbances [79]. One of the proposed mechanisms was the regulation of the cell wall mechanical properties [7], since the deposition of callose and pectins in pollen tube wall of *llg2,3* mutant was disrupted [79].

Pollen tube rupture upon reaching the embryo sac is a necessary condition for sperm release and, accordingly, for fertilization. This process is tightly controlled: recognition of the pollen tube by synergids and subsequent perception of "permitting" signal by the tube is required [8,80]. Signal peptides (for example, cysteine-rich peptides) and small molecules (for example, ROS), which allow synergid cells to recognize the pollen tube, are accumulated in the filiform apparatus (FA) area. FA is a highly thickened structure of synergids’ cell wall at the micropylar end. Pollen tube recognition at this stage is an important barrier to interspecies crossing, since “unrecognized” pollen tube does not stop growth and does not release sperms [5]. Recognition involves receptor kinase FERONIA (FER), a member of the CrRLK1L (*Catharanthus roseus* receptor-like kinase 1-like) subfamily. It regulates NORTIA (NTA) membrane anchoring and interacts with the GPI-anchored protein LORELEI (LRI) on the synergid surface [7]. Lack of recognition and of the following rupture (“overgrowth phenotype”) was found in *Arabidopsis* pistils with reduced ROS levels. In the FA area of the synergid, wild-type plants exhibit a ROS production peak, which is absent in *fer* and *lre* mutants, as well as in DPI-treated pistils. In all these cases tubes form the overgrowth phenotype and do not take part in fertilization [81]. Thus, receptor kinase FER, as well as the LRI interacting with it, are responsible for the local ROS production, which provokes tube rupture and sperm release. *abstinence by mutual consent*, *amc*, also has a phenotype similar to *fer*, but this mutant is self-sterile, that is, the phenotype is observed only when both male and female gamtophytes carry the *amc* allele. AMC encodes a peroxine involved in protein import in peroxisomes, potentially important for ROS production during pollen tube-synergid signaling [82].

## 4. Conclusions

Thus, ROS are involved in the life of the male gametophyte at all stages, from hydration to the release of sperms. On the one hand, ROS are produced endogenously, on the other, they act as a signal from female tissues. One of the main mechanisms of ROS action is the activation of ionic currents through the plasma membrane. Various ion transport systems exhibit sensitivity to ROS, but the specific pattern of their activity in each of the redox states, through which the male gametophyte passes, remains to be studied.

## Abbreviations

ROS	reactive oxygen species
ROP	Rho of plants
MnTMPP	Mn-5,10,15,20-tetrakis(1-methyl-4-pyridyl)21H,23H-porphin
CMS	cytoplasmic male sterility
DPI	diphenyl iodonium chloride
NBT	nitro blue tetrazolium
SOD	superoxide dismutase
NOX	NADPH-oxidase
MP	membrane potential
PAO	polyamine oxidase
DPI	diphenyleneiodonium (NOX inhibitor)
